# RGD-modifided oncolytic adenovirus exhibited potent cytotoxic effect on CAR-negative bladder cancer-initiating cells

**DOI:** 10.1038/cddis.2015.128

**Published:** 2015-05-14

**Authors:** Y Yang, H Xu, J Shen, Y Yang, S Wu, J Xiao, Y Xu, X-Y Liu, L Chu

**Affiliations:** 1 State Key Laboratory of Cell Biology, Institute of Biochemistry and Cell Biology, Shanghai Institutes for Biological Sciences, Chinese Academy of Sciences, Shanghai 200031, China; 2 Xinyuan Institute of Medicine and Biotechnology, Zhejiang Sci-Tech University, Hangzhou 310018, China; 3 College of Life Sciences, Northwest Agriculture and Forestry University, Yanglin 712100, China; 4 Jiangsu Center for the Collaboration and Innovation of Cancer Biotherapy, Cancer Institute, Xuzhou Medical College, Xuzhou, Jiangsu 221002, China

## Abstract

Cancer-initiating cell (CIC) is critical in cancer development, maintenance and recurrence. The reverse expression pattern of coxsackie and adenovirus receptor (CAR) and *α*_*ν*_ integrin in bladder cancer decreases the infection efficiency of adenovirus. We constructed Arg-Gly-Asp (RGD)-modified oncolytic adenovirus, carrying EGFP or TNF-related apoptosis-inducing ligand (TRAIL) gene (Onco^Ad^.RGD-hTERT-EGFP/TRAIL), and applied them to CAR-negative bladder cancer T24 cells and cancer-initiating T24 sphere cells. Onco^Ad^.RGD-hTERT-EGFP had enhanced infection ability and cytotoxic effect on T24 cells and T24 sphere cells, but little cytoxicity on normal urothelial SV-HUC-1 cells compared with the unmodified virus Onco^Ad^.hTERT-EGFP. Notably, Onco^Ad^.RGD-hTERT-TRAIL induced apoptosis in T24 cells and T24 sphere cells. Furthermore, it completely inhibited xenograft initiation established by the oncolytic adenovirus-pretreated T24 sphere cells, and significantly suppressed tumor growth by intratumoral injection. These results provided a promising therapeutic strategy for CAR-negative bladder cancer through targeting CICs.

Bladder cancer is the fourth most common cancers among men.^1^ There is a poor prognosis and 5-year survival rate of invasive bladder cancer.^2^ The risk for recurrence was significantly higher in patients with p53 nuclear accumulation^3, 4^ and abnormal pRb status.^5^ Recently, aggressive bladder cancer was reported to be associated with downregulation of coxsackie and adenovirus receptor (CAR),^6, 7, 8^ making it an interesting target for bladder cancer therapy.

One of the reasons for failure of traditional cancer therapies (such as surgery, chemotherapy or radiotherapy) is the existence of a small subpopulation in cancer, called as cancer stem (initiating) cell (CSC or CIC).^9^ Since the first application of CIC theory on leukemia in transplanted mice^10, 11^ and related experiment methods in breast cancer solid tumor about CD44^+^CD24^-^ fractions,^12^ studies have sprung up in bladder cancer.^13, 14, 15^

In our previous studies, we constructed variety of oncolytic adenoviral vectors carrying therapeutic genes and achieved potent anti-tumor effect on different types of cancers.^16^ This oncolytic viral vector-based therapy was named as 'Cancer Targeting Gene-Viro-Therapy' (CTGVT) therapeutic strategy.^17^ Our studies showed that therapeutiec genes delivered by oncolytic adenoviral vector demonstrated excellent anti-cancer effect^18, 19, 20^ and other groups have also reported that TRAIL gene elicits killing effect on CICs.^21, 22^ Adenovirus type 5 (Ad5) binds to its receptor CAR through the knob of its fiber, and internalizes into the host cell with the recognition of Arg-Gly-Asp (RGD) motif in the penton base by integrins.^23^ However, the stage- and grade-dependent CAR and integrin *α*_v_*β*_3_ expression in bladder cancer significantly influenced the infection efficacy of adenovirus and limited the application of CTGVT strategy in bladder cancer.^24, 25^ Inserting a short peptide sequence with RGD motif into the HI loop of the adenovirus knob significantly raised the infection efficacy of adenovirus.^26^ RGD-modified adenoviruses have shown potent anti-CIC effects in brain cancer.^27^ Modification on replication-associated genes and their promoters increased the replication specificity of oncolytic adenovirus in bladder cancer cells.^28^

In this work, we constructed Onco^Ad^.RGD-hTERT-EGFP, containing a RGD motif in the HI loop of fiber. Besides, adenovirus E1A region was under control of human telomerase reverse transcriptase (hTERT) promoter and the 24 base pairs pRb-binding sequence in E1A region and the E1B-55K gene were deleted. Bladder CICs were obtained through culturing bladder cancer T24 cells in specific serum-free medium. Onco^Ad^.RGD-hTERT-EGFP showed enhanced infection ability and cytotoxic effect in CAR-negative bladder cancer cells and CICs than the non-RGD modified control adenovirus, Onco^Ad^.hTERT-EGFP. Oncolytic adenovirus carrying TRAIL gene (Onco^Ad^.RGD-hTERT-TRAIL) induced apoptosis on bladder CICs and significantly inhibited initiation and growth of xenografts established by these cells. This study indicated that RGD-modified oncolytic adenovirus is a potent therapeutic way to target CAR-negative bladder cancer cells and CICs.

## Results

### T24 sphere cells possessed bladder CIC properties

**Figure 1:**
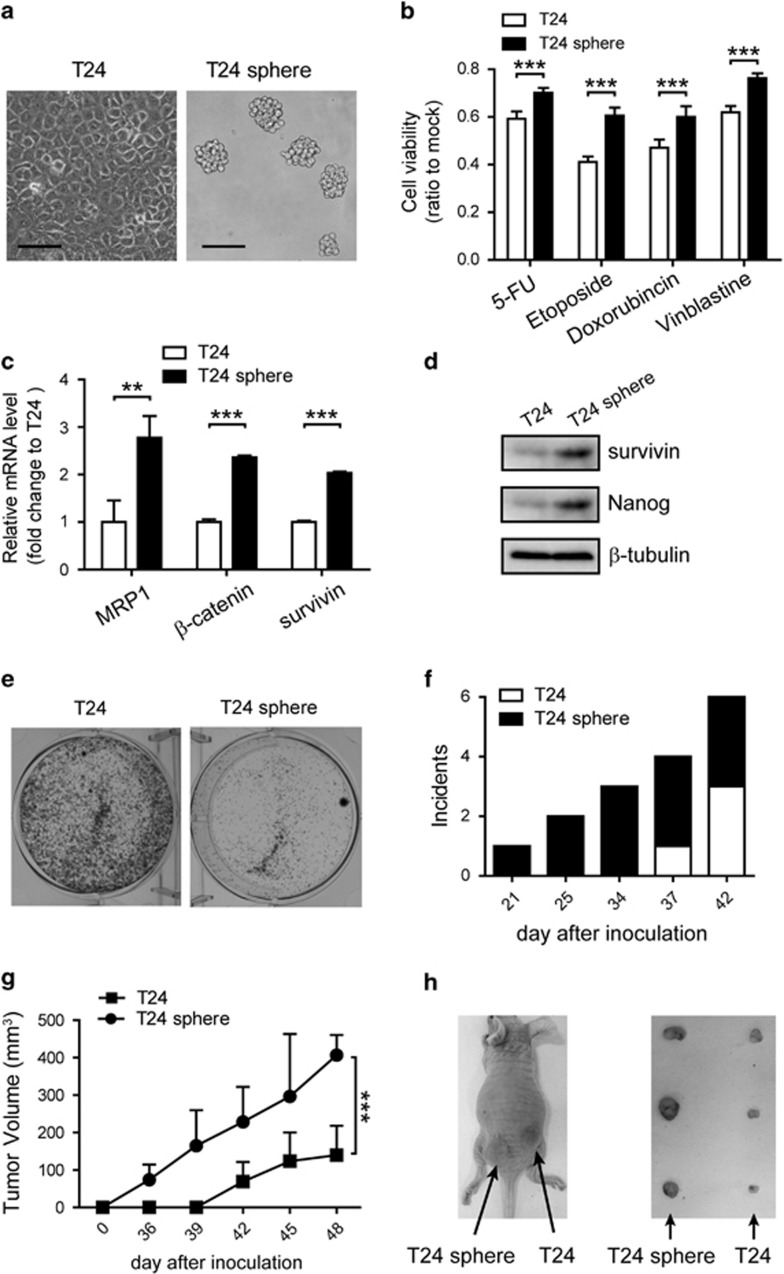
T24 sphere cells possessed bladder CIC properties. (**a**) T24 cells formed spheroid bodies 3 days after T24 cells were cultured in serum-free medium, scale bar=200 *μ*m. (**b**) T24 sphere cells displayed stronger resistance to cytotoxic chemotherapy than T24 cells after treatment with 5-FU (100 *μ*g/ml), etoposide (10 *μ*g/ml), doxorubincin (1 *μ*g/ml) and vinblastine (2 *μ*g/ml) for 2 days. Cell viability was detected with MTT assay and repeated for three times. The relative cell viability was shown by fold change to the corresponding mock. (**c**) Upregulation of *β*-catenin, survivin and MRP1 in mRNA were found in T24 sphere cells. qRT-PCR data were normalized to GAPDH gene and are shown as fold change relative to T24 cells. (**d**) Protein level of survivin and Nanog increased in T24 sphere cells. (**e**) T24 sphere cells formed smaller colonies by crystal violet staining. (**f**) T24 sphere initiated tumor earlier than T24 cells (1 × 10^3^ cells per mouse). Incidence indicated the number of mice with palpable tumor. (**g, h**) Tumor growth curve and pictures showed that T24 sphere formed larger xenografts than T24. All data shown represent mean±S.D. (*n*=3). The number of mice in each group were three. **P<0.01, ***P<0.001

Bladder cancer T24 cells were subjected to specific serum-free medium as described in materials and methods to obtain CICs. In ultra-low detachment plates, cells formed spheroid bodies and were named as T24 sphere cells (Figure 1a). To eliminate the influence of growth factor, cells were also cultured in ordinary plates, which retained adherent growth and were named as Conditioned T24 cells. A set of assays were performed to verify the properties of cancer initiating cells. Chemo-resistance of T24, T24 sphere and Conditioned T24 cells were examined by cell stability assay. T24 sphere cells presented significant resistance to cytotoxic chemotherapy compared with the other two kinds of cells (Figure 1b and Supplementary Figure S1a). The mRNA and protein levels of several genes were detected, because expression levels of genes related to self-renewal, multi-drug resistance and anti-apoptosis are usually upregulated in CICs. Elevated mRNA level of *β*-catenin, MRP1 and survivin along with protein level of survivin and Nanog were found in T24 spheres cells, compared with T24 cells (Figures 1c and d). Besides, T24 sphere cells formed smaller colonies than those of T24 cells, indicating their quiescent status (Figure 1e). As *in vivo* tumor formation ability is the golden standard for CIC,^29^ 1 × 10^3^ T24 sphere cells or T24 cells were subcutaneously injected into the left or right rear of nude mice, respectively (three mice per group). T24 sphere cells presented significantly stronger tumor-initiating ability and generated bigger tumors on nude mice (Figures 1f–h). Furthermore, after incubation in medium with serum for 6 days, the enhanced tumor-initiation ability of T24 sphere cells was compromised (five mice per group), suggesting that T24 sphere cells might possess differentiation potential (Supplementary Figures S1b–d). The above results demonstrated that T24 sphere cells maintained characters of CIC.

### RGD-modified oncolytic adenovirus exhibited enhanced infection ability and cytotoxic effect on CAR-negative T24 sphere cells

**Figure 2:**
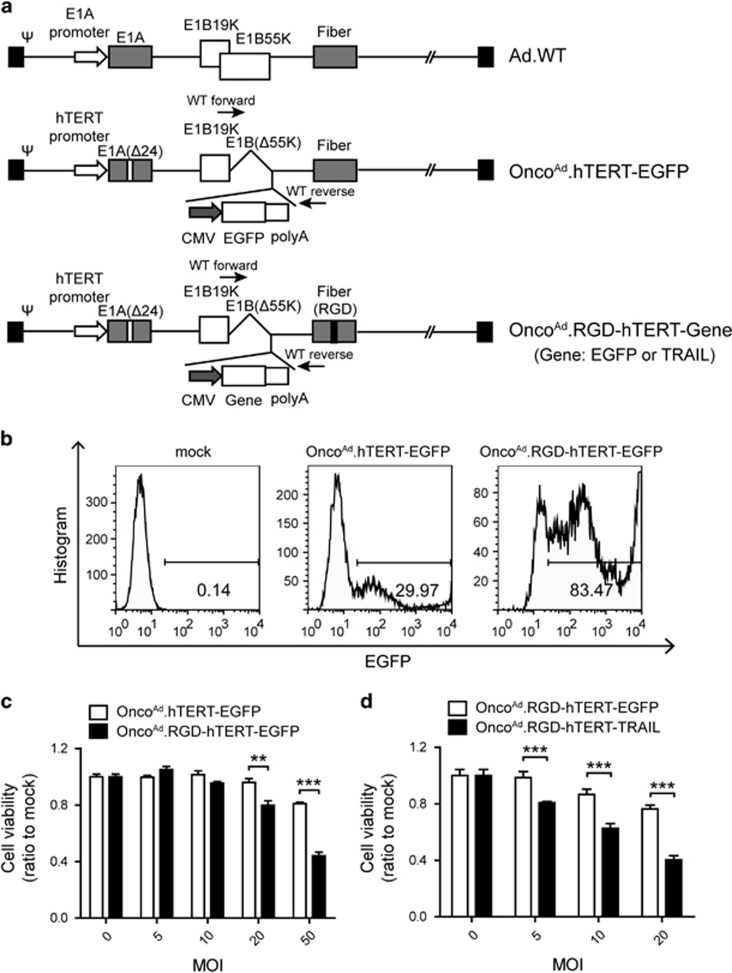
(**a**) Schematic construction of Ad genome. WT forward and reverse represented the primer location for identification of wide-type contamination. (**b**) The proportion of EGFP-positive cells were significantly increased in T24 sphere cells treated with Onco^Ad^.RGD-hTERT-EGFP for 2 days than cells treated with Onco^Ad^.hTERT-EGFP. EGFP-positive cells were detected by fluorescence-activated cell sorting analysis. (**c**) Comparison on cell viability of T24 sphere cells treated with Onco^Ad^.hTERT-EGFP and Onco^Ad^.RGD-hTERT-EGFP at indicated MOI for 4 days. (**d**) Examination of 2-day cytotoxicity elicited on T24 sphere cells infected with Onco^Ad^.RGD-hTERT-EGFP and Onco^Ad^.RGD-hTERT-TRAIL. Cell viability was determined by MTT assay. All the experiments were repeated three times and all data shown represented mean±S.D. (*n*=3). **P<0.01, ***P<0.001

As T24 sphere cells were CAR-negative cells (Supplementary Figure S1e), which influenced infection efficacy of adenovirus, modifications are demanded for enhanced infection ability. RGD-modified oncolytic adenovirus Onco^Ad^.RGD-hTERT-EGFP and Onco^Ad^.RGD-hTERT-TRAIL as well as the control were constructed (Figure 2a). The RGD-modified virus contained a short sequence encoding CDCRGDCFC (RGD-4C) peptide in the HI loop of fiber coding region. EGFP or TRAIL gene was inserted in the E1B-55K deletion region. Oncolytic adenoviruses were packaged and amplified in HEK-293 cells. PCR amplification and sequencing of PCR products were used to confirm insertion of RGD motif in fiber region and deletion of 24 bp in E1A (Supplementary Figure S2a). Virus stocks were demonstrated free of wild-type adenovirus and E1B-55K gene deletion according to the different length between E1B-55K gene and inserted gene (EGFP or TRAIL) (Supplementary Figure S2b).

To investigate the infection ability and cytotoxicity of RGD-modified oncolytic adenovirus on bladder CICs, T24 sphere cells were infected in monolayer or spheroid status, respectively, with indicated MOI of Onco^Ad^.RGD-hTERT-EGFP, and Onco^Ad^.hTERT-EGFP served as non-RGD modification control. Larger proportion of EGFP-positive cells were observed in T24 sphere cells treated with Onco^Ad^.RGD-hTERT-EGFP (Figure 2b and Supplementary Figures S2c–e), implying the superior infection ability of RGD-modified oncolytic adenovirus. RGD-modified viruses exhibited higher infection efficiency than non-modified control by absolute quantitation of adenovirus genomic DNA with real-time qPCR (Supplymentary Figure S3a and Supplymentary Table S2). Onco^Ad^.RGD-hTERT-EGFP also presented advanced proliferation inhibition effect on T24 sphere cells (Figure 2c), and the effect was further increased after carrying TRAIL gene (Figure 2d and Supplementary Figures S3b and c). Additionally, although Onco^Ad^.RGD-hTERT-EGFP exhibited stronger infection action on bladder cancer T24 cells and normal uroepithelial SV-HUC-1 cells, it only significantly inhibited T24 cell growth (Supplementary Figure S4), indicating the replication specificity of RGD-modified oncolytic adenovirus in cancer cells.

### Onco^Ad^.RGD-hTERT-TRAIL induced T24 sphere cells apoptosis

**Figure 3:**
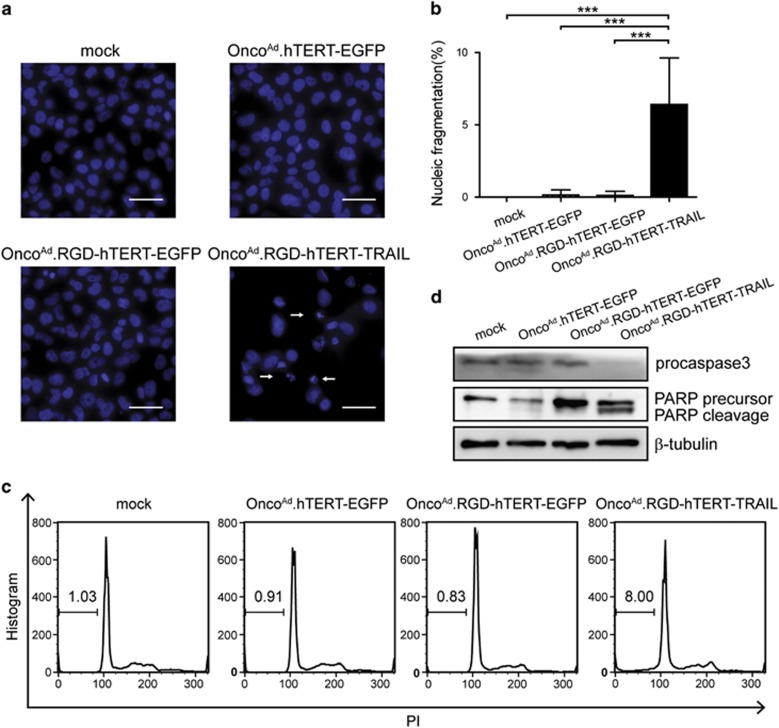
RGD-modified oncolytic adenovirus exhibited enhanced infection ability and elicited cytotoxic effect on CAR-negative T24 sphere cells by inducing cell apoptosis. (**a**) Increased nucleic fragmentation (arrow) was observed in T24 sphere cells after 2-day treatment of Onco^Ad^.RGD-hTERT-TRAIL (10 MOI) as detected by Hoechst staining, scale bar: 100 *μ*m. (**b**) Statistic data for three repeats of (**a**). The percentage (%)=Number of nucleic fragmented cells in six fields/Number of total cells in six fields. (**c**) Two-day Onco^Ad^.RGD-hTERT-TRAIL (10 MOI) treatment increased Sub-G1 population of T24 sphere cells. (**d**) Upregulation of PARP protein cleavage and downregulation of procaspase3 protein were observed in T24 sphere cells after treatment with Onco^Ad^.RGD-hTERT-TRAIL (10 MOI) for 2 days. All the experiments were repeated three times and all data shown represented mean±S.D. (*n*=3). ***P<0.001

We next determined whether Onco^Ad^.RGD-hTERT-TRAIL induced apoptosis on T24 sphere cells. Hoechst 33258 staining and flow cytometry assay disclosed that the fraction of nucleic fragmentation and sub-G1 phase of T24 sphere cells raised significantly after Onco^Ad^.RGD-hTERT-TRAIL treatment (Figures 3a–c). Decreased protein level of pro-caspase 3 and increased cleavage form of PARP protein were exposed by western blot, indicating that Onco^Ad^.RGD-hTERT-TRAIL induced T24 sphere cells apoptosis via caspase-dependent pathway (Figure 3d). Notably, Onco^Ad^.RGD-hTERT-TRAIL can also induce caspase-dependent apoptosis in T24 cells (Supplementary Figure S5).

### Onco^Ad^.RGD-hTERT-TRAIL suppressed tumor initiation and growth *in vivo*

**Figure 4:**
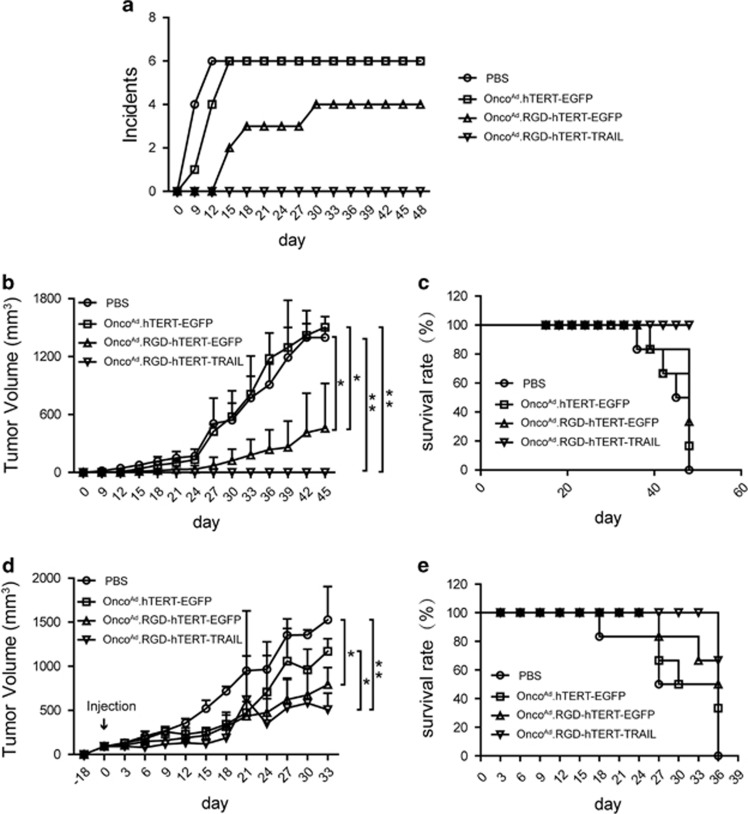
Onco^Ad^.RGD-hTERT-TRAIL suppressed tumor initiation and growth *in vivo*. (**a**) Comparison of tumor-initiating ability on T24 sphere cells pre-treated with PBS, Onco^Ad^.hTERT-EGFP, Onco^Ad^.RGD-hTERT-EGFP and Onco^Ad^.RGD-hTERT-TRAIL for 4 h. The number of tumor occurrence was plotted against time after inoculation (day 0). Incidence indicated the number of mice with palpable tumor. (**b**) Growth curve of subcutaneous tumors with the indicated pre-treatment. (**c**) Survival status of mice with the indicated pre-treatment. (**d**) Growth curve of subcutaneous tumors with the indicated intra-tumor adenovirus injection. Oncolytic adenoviruses were intratumorally injected (day 0) after subcutaneous tumor inoculated for 18 days, with corresponding volume of PBS control. Arrow represented the action of intratumoral injection. (**e**) Survival status of mice with the indicated intratumoral treatment. Tumor volume was measured every 3 days and the data shown represented mean±S.D. (*n*=6). The number of mice for each kind of treatment was six. *P <0.05, **P<0.01

As CICs usually hold strong tumorigenecity, subcutaneous xenograft models on nude mice were established by T24 sphere cells to test tumor suppression capacity of Onco^Ad^.RGD-hTERT-TRAIL *in vivo* (six mice per group). T24 sphere cells pre-infected with Onco^Ad^.RGD-hTERT-TRAIL failed to form xenografts, and Onco^Ad^.RGD-hTERT-EGFP pre-treatment resulted in initiation latency and significantly slower growth rate (Figures 4a and b). Prolonged survival rate was observed in groups treated with RGD-modified virus, as compared with the control mice (Figure 4c). Although Onco^Ad^.RGD-hTERT-EGFP and Onco^Ad^.RGD-hTERT-TRAIL did not significantly improve the survival of mice through intratumoral injection, both of them repressed growth of xenograft established by T24 spheres to nearly the same extent (six mice per group) (Figures 4d and e).

## Discussion

In this study, RGD-modified and multi-regulated oncolytic adenovirus named Onco^Ad^.RGD-hTERT-TRAIL was constructed and displayed potent cytotoxic effect in bladder CICs. Modification of this adenovirus included: RGD motif containing peptide sequence insertion in fiber; hTERT promoter driving E1A gene; deletion of Rb-binding domain of E1A and E1B-55K; and carrying TRAIL expression cassette (Figure 2a).

Owing to the reverse expression pattern of CAR and integrin in bladder cancer, adenovirus infection efficiency was severely suppressed. RGD modification significantly increased the ability of adenovirus entering into CAR-deficient bladder cancer cells.^30, 31, 32^ hTERT promoter-driven oncolytic adenovirus increased clinical safety.^33^ Modifications can be made to improve the replication specificity of adenovirus through targeting Rb and p53 abnormalities which lead to poor outcome in bladder cancer patients.^34^ TRAIL-armed oncolytic adenovirus had excellent performance on stem-like esophageal cancer cells and plenty of other types of cancer.^18, 21, 22^

Different types of adenoviruses were utilized and modified for gene transfer and targeted therapy on bladder cancer.^35, 36^ However, as for the emerging evidence of bladder CICs in bladder, it is important to find out efficient oncolytic adenoviruses targeting bladder CICs. We designed the RGD-modified oncolytic adenoviruses to overcome CAR deficiency in bladder cancer and examined their therapeutic potential on bladder CICs.

It is crucial to obtain CICs. Pathological data indicated that bladder cancer invasiveness was linked with less differentiated status,^37^ supporting the CIC hypothesis and providing biological and molecular clues for isolation and identification of CICs. Prevalently, cell sorting by flow cytometry and accumulation through specific culture condition were the main two methods for CIC isolation.^38, 39^ Cell sorting substantially depends on cell surface markers, cytokeratins, side populations and aldehyde dehydrogenase.^40^ Cell surface proteins such as CD44 and its variant CD44v6 was developed as potential markers for CICs and cytokeratin 5 were reported to have properties of CICs.^41, 42^ Side population separated from bladder cancer cells showed self-renewal and differentiation characters. Besides, aldehyde dehydrogenase 1 A1 (ALDHA1) activity was a promising choice for bladder CIC accumulation.^43^ However, sorting markers were not quite consistent among different clinical samples and cell lines, causing the complexity and flexibility of identification process.^44^ In our work, bladder cancer-initiating T24 sphere cells were gained from specific culture condition and identified to possess CIC characters, including chemo-resistance, self-renewal, quiescence, differentiation and tumor initiation (Figure 1 and Supplementary Figure S1).

Traditional therapeutic strategies confronted failure on killing CIC, which displayed high level of multi-drug resistant gene expression and expelling small molecule capacity.^45^ Adenovirus infected cells through its own pathway and won't be pumped out of CICs, implying its potential function in CIC targeted therapy.^46^ However, its infection efficiency closely related to CAR expression level, which restricted its effect on CAR deficient cells. Here, enhanced infection ability and cell growth repression on T24 sphere cells were observed after treatment of the constructed oncolytic adenovirus, Onco^Ad^.RGD-hTERT-EGFP (Figures 2b and c). TRAIL gene-armed adenovirus Onco^Ad^.RGD-hTERT-TRAIL significantly reduced T24 sphere cell growth *in vitro* and xenograft initiation and progression *in vivo* (Figures 3d and 4). Notably, Onco^Ad^.RGD-hTERT-EGFP elicited cytotoxic effect on bladder cancer T24 cells while had little influence on normal urinary epithelial SV-HUC-1 cells (Supplementary Figures S4c and d), which is in accordance with the *in vivo* results. These results indicated that RGD-modified oncolytic adenovirus with therapeutic genes is a promising strategy for bladder cancer therapy and might reduce risk of recurrence.

In addition, the *in vivo* anti-tumor effect of our CTGVT therapeutic strategy depends on the carried gene expression and oncolytic adenovirus itself. TRAIL protein needs to be secreted out of cells and delivered to other cells to continue its function, which was influenced by injection dose of virus, immune response and the complex microenvironment *in vivo*. These might together cause the discrepancy between our *in vitro* and *in vivo* results, and contribute to the lack of effect observed on tumor suppression level and survival. However, this could potentially be improved by combining our virus with other chemotherapeutics or interferon, as TRAIL is capable of increasing chemo-sensitivity and acting synergistically with interferon-alpha. Besides, considering the unique characters of bladder CICs, further modifications might be essential. Utilization of bladder cancer-specific promoter such as survivin promoter^47^ or human Uroplakin II (UPII) promoter^35^ for E1A control achieved enhanced replication selectivity in bladder cancer. The therapeutic gene can be replaced with shRNAs, miRNAs or monoclonal antibodies targeting CIC self-renewal or differentiation pathway. Furthermore, whether combination of two oncolytic adenoviruses carrying different genes or delivering two genes by single virus will achieve enhanced targeting effect on bladder CICs also remains to be testified.

In conclusion, our data showed that the constructed Onco^Ad^.RGD-hTERT-TRAIL exhibited robust effect in inhibition bladder cancer initiating cells *in vitro* and *in vivo*, suggesting the potential anti-tumor possibility for bladder cancer therapy.

## Materials and Methods

### Cell culture and reagents

The human bladder cancer T24 cells and normal uroepithelial SV-HUC-1 cells were obtained from Cell Bank of the Type Culture Collection of Chinese Academy of Sciences (Shanghai, China) and incubated in McCoy's 5A or F-12K medium supplemented with 10 or 20% heat-inactivated fetal bovine serum, respectively, at 37 °C in a humidified atmosphere with 5% CO_2_.

T24 cells were incubated with serum-free DMEM/F12 (Hyclone, Waltham, MA, USA) medium in ultra-low attachment 6-well dishes (Corning, Tewksbury, MA, USA). Growth factors including EGF, b-FGF and IGF-1 were supplied at a concentration of 20 ng/ml (PeproTech, Rocky Hill, NJ, USA) each day (T24 sphere cells). Three days after seeding, the propagated spheroid bodies were collected and digested by StemPro Accutase (Thermo Fisher, Waltham, MA, USA) to single cell suspension for subsequent experiments except for 3D infection test, in which they were directly used. The same medium and growth factors were utilized for T24 cells in ordinary 6-well dishes (Conditioned T24 cells).

### Adenoviruses construction and identification

The CDCRGDCFC coding sequence were inserted into adenovirus backbone plasmid (pAdeasy-1-E3) by overlap PCR utilizing the two pairs of RGD-related primers described in Supplementary Table S1. The expression cassette of EGFP and TRAIL were inserted into the E1B-55K-deleted region of a shuttle vector (pShuttle-hTERT-E1A(Δ24)-E1B(Δ55)). Different oncolytic adenoviruses plasmids were generated through homologous recombination of the shuttle vector and adenoviral backbone plasmid in *E. coli.*BJ5183 cells.^48^ Then, viruses were packaged and amplified in HEK-293 cells, followed by gradient CsCl solution centrifugation for purification. Titer was measured by QuickTiter Adenovirus Titer Immunoassay Kit (Cell Biolabs, San Diego, CA, USA); the IFU/PFU ratio for Onco^Ad^.hTERT-EGFP, Onco^Ad^.RGD-hTERT-EGFP and Onco^Ad^.RGD-hTERT-TRAIL was 1.71, 1.75 and 2.30, respectively. Virus genomes were extracted according to the protocol of Blood Genome Extract Kit (Generay, Shanghai, China). The existence of peptide RGD coding sequence and 24 bp deletion, together with the E1B-55K deletion and wild-type contamination were demonstrated by PCR and sequencing with corresponding primers (Supplementary Table S1). The expression of TRAIL gene was examined by western blot.

### Chemo-resistance, colony formation and cell cycle analysis

MTT assay was used to measure chemo-resistance. After seeding at a density of 1 × 10^3^ cells per well in the 96-well plates for 12 h, cells were treated with 5-FU, etoposide, doxorubicin or vinblastine. Cell viability was determined 48 h later. Cells in each well were incubated with 20 *μ*l 3-(4,5-dimethylthiazol-2-yl)-2,5-diphenyltetrazolium bromide (MTT, Beyotime, China) at 37 °C for 4 h. Supernatants were discarded and 100 *μ*l dimethyl sulfoxide (Guanghua Sci-Tech, China) was added into each well to dissolve the remains. Dual wavelength of 595 and 650 nm were applied in absorbance assessment via a Microplate Reader (Thermo Fisher). As for colony formation assay, T24 cells and T24 sphere cells were seeded at a density of 1 × 10^3^ cells per well in 6-well plates for 6 days. Then, colonies after fixation and subsequently crystal violet staining were photographed. Cell cycle analysis were reflected by propidium iodide staining. Cells were digested and resuspended in 200 ml PBS. After fixation in 70% ethanol overnight at 4 °C for 4 h, cells were treated with RNase A (Generay, China) (20 *μ*g/ml) at 37 °C for 30 min, and stained with 50 *μ*g/ml propidium iodide (Sigma, St. Louis, MO, USA) for another 30 min. Fluorescence-activated cell sorting was used for data acquirement. All experiments were repeated for three times.

### Gene expression assay

Quantitative RT-PCR (qRT-PCR) assay was accomplished with Trizol (Invitrogen, Carlsbad, CA, USA) for total RNA isolation, ReverTra Ace qPCR RT Kit (Toyobo, Japan) for reverse transcription and SYBR Green Realtime PCR Master Mix (Toyobo, Japan) for quantitation, according to the corresponding protocols. mRNA expression levels of MRP1, survivin and *β*-catenin were evaluated by their specified primers (Supplementary Table S1) with GAPDH as an internal control. Each assay was done in triplicate.

Primary antibodies against Nanog and survivin (Cell Signaling Technology, Danvers, MA, USA), caspase 8, caspase 3, PARP and TRAIL (Santa Cruz biotechnology, Santa Cruz, CA, USA) along with GAPDH (CoWin Bioscience, Bejing, China) were used for western blot. All the secondary antibodies were purchased from Santa Cruz biotechnology. CAR expression level were detected by fluorescence-activated cell sorting with PE-conjugated primary antibody against CAR (Millpore, Billerica, MA, USA) and mouse IgG-1 as isotype control (BD, Franklin Lakes, NJ, USA)

### Virus infection ability and efficiency detection

T24, T24 sphere and SV-HUC-1 cells were infected with Onco^Ad^.hTERT-EGFP or Onco^Ad^.RGD-hTERT-EGFP at indicated MOI for 48 h, respectively, for ordinary infection ability analysis. In addition, spheroids were mixed with 4 × 10^6^ IFU virus (Onco^Ad^.hTERT-EGFP or Onco^Ad^.RGD-hTERT-EGFP, respectively) and cultured for 48 h to testify the 3D infection ability. EGFP-positive cells were photographed and quantified by fluorescence microscope and fluorescence-activated cell sorting.

To test infection efficiency, T24 sphere cells (2 × 10^5^) were infected with indicated adenoviruses at 20 MOI for 6 h and harvested after washing by PBS for three times. Total genomic DNA were extracted from these cells utilizing QIAamp DNA Mini Kit (QIAGEN, Dusseldorf, Germany) and the copy number of virus genome were determined by real-time PCR of E3 gene with absolute quantitation method (primers were described in Supplementary Table S1). Standard curves were drawn according to corresponding pure virus genomic DNA. Copy Number=( amount × 6.02 × 10^23^)/(length × 1 × 10^9^ × 660); Efficiency of infection=Copy Number of virus genome/2 × 10^5^.

### Hochst33258 staining

T24 sphere cells were infected with Onco^Ad^.hTERT-EGFP, Onco^Ad^.RGD-hTERT-EGFP and Onco^Ad^.RGD-hTERT-TRAIL at a MOI of 20 for 48 h. Cells were fixed with 4% paraformaldehyde (Sigma) for 15 min and stained with Hoechst33258 (Molecular Probes, Eugene, OR, USA) at 1 *μ*g/ml for 1 min, and subjected to fluorescence microscope.

### Animal experiments

All the animal experiments were approved by the Institutional Animal Care and Use Committee, and performed according to the U.S. Public Health Service Policy on Humane Care and the Use of Laboratory Animals. Four-week-old female BALB/c nude mice were purchased from SLAC (Shanghai, China) and raised in the IVC animal facilities of Zhejiang Chinese Medical University.

In tumorigenecity assay, T24 cells and T24 sphere cells were mixed with matrigel (BD) at 2:1, and subcutaneously injected into the right and left rear back of mice, respectively. Total 1 × 10^3^ cells were injected into each mouse, and each group included three mice. T24 sphere cells were cultured in ordinary medium for 6 days and named as cultured T24 sphere cells. These cultured sphere cells were injected at 1 × 10^2^ cells per mouse in the above method to observe the differentiation potential of T24 sphere cells. Same amount of T24 and T24 sphere cells were used as control. Each group included five mice. The status of tumor occurrence was observed every other day.

To determine the *in vivo* anti-CIC effect of adenoviruses on T24 sphere xenografts, 2 × 10^5^ T24 sphere cells were pre-incubated with 100 *μ*l PBS or 5 MOI viruses (Onco^Ad^.hTERT-EGFP, Onco^Ad^.RGD-hTERT-EGFP and Onco^Ad^.RGD-hTERT-TRAIL) for 4 h and subcutaneously injected into each mouse with Matrigel at 2:1. Each group included six mice. Further, 2 × 10^5^ T24 sphere cells were injected at the right rear of nude mice with Matrigel at 2:1. After the tumor volume reached around 90 mm^3^, mice were randomly divided into four groups (six mice each) and intratumorally treated with 100 ml PBS or 5 × 10^8^ IFU viruses (Onco^Ad^.hTERT-EGFP, Onco^Ad^.RGD-hTERT-EGFP and Onco^Ad^.RGD-hTERT-TRAIL) twice at a 1-day interval. The tumor volume were measured every 2 days and calculated as length × width × width/2.

### Statistical analysis

All the data were shown as mean±S.D. or mean+.S.D. Comparison between groups were performed by student's *t*-test or one-way analysis of variance using R software.

## Abbreviations

Ad5: Adenovirus type 5
ALDHA1: aldehyde dehydrogenase 1 A1
CAR: coxsackie virus and adenovirus receptor
CSC (CIC): cancer stem (initiating) cell
CTGVT: Cancer Targeting Gene-Viro-Therapy
hTERT: human telomerase reverse transcriptase
MRP1: multidrug resistance protein 1
MTT: 3-(4, 5-dimethylthiazol-2-yl)-2, 5-diphenyl tetrazolium bromide
PBS: phosphate buffered saline
RGD: Arg-Gly-Asp
TRAIL: TNF-related apoptosis-inducing ligand
WT: wild type

## References

[bib1] Siegel R, Ma JM, Zou ZH, Jemal A. Cancer Statistics, 2014. CA Cancer J Clin 2014; 64: 9–29. 24399786 10.3322/caac.21208

[bib2] Hassen W, Droller MJ. Current concepts in assessment and treatment of bladder cancer. Curr Opin Urol 2000; 10: 291–299. 10918966 10.1097/00042307-200007000-00002

[bib3] Esrig D, Elmajian D, Groshen S, Freeman JA, Stein JP, Chen SC et al. Accumulation of nuclear p53 and tumor progression in bladder cancer. N Engl J Med 1994; 331: 1259–1264. 7935683 10.1056/NEJM199411103311903

[bib4] Cote RJ, Esrig D, Groshen S, Jones PA, Skinner DG. p53 and treatment of bladder cancer. Nature 1997; 385: 123–125. 10.1038/385123b0 8990112

[bib5] Benedict WF, Lerner SP, Zhou J, Shen X, Tokunaga H, Czerniak B. Level of retinoblastoma protein expression correlates with p16 (MTS-1/INK4A/CDKN2) status in bladder cancer. Oncogene 1999; 18: 1197–1203. 10022125 10.1038/sj.onc.1202452

[bib6] Matsumoto K, Shariat SF, Ayala GE, Rauen KA, Lerner SP. Loss of coxsackie and adenovirus receptor expression is associated with features of aggressive bladder cancer. Urology 2005; 66: 441–446. 16040097 10.1016/j.urology.2005.02.033

[bib7] Okegawa T, Pong RC, Li Y, Bergelson JM, Sagalowsky AI, Hsieh JT. The mechanism of the growth-inhibitory effect of coxsackie and adenovirus receptor (CAR) on human bladder cancer: a functional analysis of car protein structure. Cancer Res 2001; 61: 6592–6600. 11522659

[bib8] Sachs MD, Rauen KA, Ramamurthy M, Dodson JL, De Marzo AM, Putzi MJ et al. Integrin alpha(v) and coxsackie adenovirus receptor expression in clinical bladder cancer. Urology 2002; 60: 531–536. 12350512 10.1016/s0090-4295(02)01748-x

[bib9] Zhou BB, Zhang H, Damelin M, Geles KG, Grindley JC, Dirks PB. Tumour-initiating cells: challenges and opportunities for anticancer drug discovery. Nat Rev Drug Discov 2009; 8: 806–823. 19794444 10.1038/nrd2137

[bib10] Lapidot T, Sirard C, Vormoor J, Murdoch B, Hoang T, Caceres-Cortes J et al. A cell initiating human acute myeloid leukaemia after transplantation into SCID mice. Nature 1994; 367: 645–648. 7509044 10.1038/367645a0

[bib11] Bonnet D, Dick JE. Human acute myeloid leukemia is organized as a hierarchy that originates from a primitive hematopoietic cell. Nat Med 1997; 3: 730–737. 9212098 10.1038/nm0797-730

[bib12] Al-Hajj M, Wicha MS, Benito-Hernandez A, Morrison SJ, Clarke MF. Prospective identification of tumorigenic breast cancer cells. Proc Natl Acad Sci USA 2003; 100: 6890–6890. 10.1073/pnas.0530291100 PMC153034 12629218

[bib13] Yang YM, Chang JW. Bladder cancer initiating cells (BCICs) are among EMA-CD44v6+ subset: novel methods for isolating undetermined cancer stem (initiating) cells. Cancer Invest 2008; 26: 725–733. 18608209 10.1080/07357900801941845

[bib14] Falso MJ, Buchholz BA, White RW. Stem-like cells in bladder cancer cell lines with differential sensitivity to cisplatin. Anticancer Res 2012; 32: 733–738. 22399585 PMC3638955

[bib15] Ojha R, Jha V, Singh SK, Bhattacharyya S. Autophagy inhibition suppresses the tumorigenic potential of cancer stem cell enriched side population in bladder cancer. Biochim Biophys Acta 2014; 1842: 2073–2086. 25020236 10.1016/j.bbadis.2014.07.007

[bib16] Xin-Yuan L, Huang W-L, Qian Q-J, Zou W-G, Zhang Z-L, Chu L et al. 2 - Cancer targeting gene–viro–therapy and its promising future: A trend in both cancer gene therapy and cancer virotherapy. In: Shi X-YLP-F (ed), Recent Advances in Cancer Research and Therapy. Elsevier: : Oxford, 2012 pp 33–83.

[bib17] Liu XY. Targeting gene-virotherapy of cancer and its prosperity. Cell Res 2006; 16: 879–886. 17102812 10.1038/sj.cr.7310108

[bib18] Pei Z, Chu L, Zou W, Zhang Z, Qiu S, Qi R et al. An oncolytic adenoviral vector of Smac increases antitumor activity of TRAIL against HCC in human cells and in mice. Hepatology 2004; 39: 1371–1381. 15122766 10.1002/hep.20203

[bib19] Zhao L, Dong A, Gu J, Liu Z, Zhang Y, Zhang W et al. The antitumor activity of TRAIL and IL-24 with replicating oncolytic adenovirus in colorectal cancer. Cancer Gene Ther 2006; 13: 1011–1022. 16799468 10.1038/sj.cgt.7700969

[bib20] Xu HN, Shen ZX, Xiao J, Yang Y, Huang WD, Zhou ZM et al. Acetylcholinesterase overexpression mediated by oncolytic adenovirus exhibited potent anti-tumor effect. BMC Cancer 2014; 14: 668. 25220382 10.1186/1471-2407-14-668 PMC4169801

[bib21] Zhang X, Komaki R, Wang L, Fang B, Chang JY. Treatment of radioresistant stem-like esophageal cancer cells by an apoptotic gene-armed, telomerase-specific oncolytic adenovirus. Clin Cancer Res 2008; 14: 2813–2823. 18451249 10.1158/1078-0432.CCR-07-1528 PMC2387204

[bib22] Loebinger MR, Sage EK, Davies D, Janes SM. TRAIL-expressing mesenchymal stem cells kill the putative cancer stem cell population. Br J Cancer 2010; 103: 1692–1697. 21063402 10.1038/sj.bjc.6605952 PMC2994223

[bib23] Berk AJ. Fields Virology, vol. 2. Lippincott Williams & Wilkins: : Philadelphia, 2007.

[bib24] Wang H, Cai Z, Yang F, Luo J, Satoh M, Arai Y et al. Enhanced antitumor efficacy of integrin-targeted oncolytic adenovirus AxdAdB3-F/RGD on bladder cancer. Urology 2014; 83: e513–509. 10.1016/j.urology.2013.10.025 24315309

[bib25] Li Y, Pong RC, Bergelson JM, Hall MC, Sagalowsky AI, Tseng CP et al. Loss of adenoviral receptor expression in human bladder cancer cells: a potential impact on the efficacy of gene therapy. Cancer Res 1999; 59: 325–330. 9927041

[bib26] Krasnykh V, Dmitriev I, Mikheeva G, Miller CR, Belousova N, Curiel DT. Characterization of an adenovirus vector containing a heterologous peptide epitope in the HI loop of the fiber knob. J Virol 1998; 72: 1844–1852. 9499035 10.1128/jvi.72.3.1844-1852.1998 PMC109474

[bib27] Jiang H, Gomez-Manzano C, Aoki H, Alonso MM, Kondo S, McCormick F et al. Examination of the therapeutic potential of Delta-24-RGD in brain tumor stem cells: role of autophagic cell death. J Natl Cancer Inst 2007; 99: 1410–1414. 17848677 10.1093/jnci/djm102

[bib28] Lanson NA Jr., Friedlander PL, Schwarzenberger P, Kolls JK, Wang G. Replication of an adenoviral vector controlled by the human telomerase reverse transcriptase promoter causes tumor-selective tumor lysis. Cancer Res 2003; 63: 7936–7941. 14633724

[bib29] Welte Y, Adjaye J, Lehrach HR, Regenbrecht CRA. Cancer stem cells in solid tumors: elusive or illusive? Cell Commun Signal 2010; 8: 6. 20459772 10.1186/1478-811X-8-6 PMC2880310

[bib30] Borovjagin AV, Krendelchtchikov A, Ramesh N, Yu DC, Douglas JT, Curiel DT. Complex mosaicism is a novel approach to infectivity enhancement of adenovirus type 5-based vectors. Cancer Gene Ther 2005; 12: 475–486. 15706356 10.1038/sj.cgt.7700806

[bib31] Wakayama M, Abei M, Kawashima R, Seo E, Fukuda K, Ugai H et al. E1A, E1B double-restricted adenovirus with RGD-fiber modification exhibits enhanced oncolysis for CAR-deficient biliary cancers. Clin Cancer Res 2007; 13: 3043–3050. 17505007 10.1158/1078-0432.CCR-06-2103

[bib32] Hiwasa K, Nagaya H, Terao S, Acharya B, Hamada K, Mizuguchi H et al. Improved gene transfer into bladder cancer cells using adenovirus vector containing RGD motif. Anticancer Res 2012; 32: 3137–3140. 22843884

[bib33] Nemunaitis J, Tong AW, Nemunaitis M, Senzer N, Phadke AP, Bedell C et al. A phase I study of telomerase-specific replication competent oncolytic adenovirus (telomelysin) for various solid tumors. Mol Ther 2010; 18: 429–434. 19935775 10.1038/mt.2009.262 PMC2839300

[bib34] Singh A, Jones RF, Friedman H, Hathir S, Soos G, Zabo A et al. Expression of p53 and pRb in bladder and prostate cancers of patients having both cancers. Anticancer Res 1999; 19: 5415–5417. 10697570

[bib35] Wang L, Zhang Y, Zhao J, Xiao E, Lu J, Fu S et al. Combination of bladder cancer-specific oncolytic adenovirus gene therapy with cisplatin on bladder cancer *in vitro*. Tumour Biol 2014; 35: 10879–10890. 25085582 10.1007/s13277-014-2353-7

[bib36] Melquist JJ, Kacka M, Li YM, Malaeb BS, Elmore J, Baseman AG et al. Conditionally replicating adenovirus-mediated gene therapy in bladder cancer: An orthotopic *in vivo* model. Urol Oncol 2006; 24: 362–371. 16818192 10.1016/j.urolonc.2005.11.028

[bib37] Fujiyama C, Jones A, Fuggle S, Bicknell R, Cranston D, Harris AL. Human bladder cancer invasion model using rat bladder *in vitro* and its use to test mechanisms and therapeutic inhibitors of invasion. Br J Cancer 2001; 84: 558–564. 11207054 10.1054/bjoc.2000.1641 PMC2363759

[bib38] Keymoosi H, Gheytanchi E, Asgari M, Shariftabrizi A, Madjd Z. ALDH1 in combination with CD44 as putative cancer stem cell markers are correlated with poor prognosis in urothelial carcinoma of the urinary bladder. Asian Pac J Cancer Prev 2014; 15: 2013–2020. 24716927 10.7314/apjcp.2014.15.5.2013

[bib39] Na YR, Seok SH, Kim DJ, Han JH, Kim TH, Jung H et al. Isolation and characterization of spheroid cells from human malignant melanoma cell line WM-266-4. Tumor Biol 2009; 30: 300–309. 10.1159/000261073 19940551

[bib40] Ho PL, Kurtova A, Chan KS. Normal and neoplastic urothelial stem cells: getting to the root of the problem. Nat Rev Urol 2012; 9: 583–594. 22890301 10.1038/nrurol.2012.142 PMC3468664

[bib41] Yang YM, Chang JW. Bladder cancer initiating cells (BCICs) are among EMA(-)CD44v6(+) subset: Novel methods for isolating undetermined cancer stem (initiating) cells. Cancer Investig 2008; 26: 725–733. 18608209 10.1080/07357900801941845

[bib42] Shin K, Lim A, Odegaard JI, Honeycutt JD, Kawano S, Hsieh MH et al. Cellular origin of bladder neoplasia and tissue dynamics of its progression to invasive carcinoma. Nat Cell Biol 2014; 16: 469–U194. 24747439 10.1038/ncb2956 PMC4196946

[bib43] Falso MJS, Buchholz BA, White RWD. Stem-like cells in bladder cancer cell lines with differential sensitivity to cisplatin. Anticancer Res 2012; 32: 733–738. 22399585 PMC3638955

[bib44] Klatte T, Seligson DB, Rao JY, Yu H, de Martino M, Garraway I et al. Absent CD44v6 expression is an independent predictor of poor urothelial bladder cancer outcome. J Urol 2010; 183: 2403–2408. 20403620 10.1016/j.juro.2010.01.064

[bib45] Dean M, Fojo T, Bates S. Tumour stem cells and drug resistance. Nat Rev Cancer 2005; 5: 275–284. 15803154 10.1038/nrc1590

[bib46] Short JJ, Curiel DT. Oncolytic adenoviruses targeted to cancer stem cells. Mol Cancer Ther 2009; 8: 2096–2102. 19671759 10.1158/1535-7163.MCT-09-0367

[bib47] Seo HK, Seo JB, Nam JK, Jeong KC, Shin SP, Kim IH et al. Development of replication-competent adenovirus for bladder cancer by controlling adenovirus E1a and E4 gene expression with the survivin promoter. Oncotarget 2014; 5: 5615–5623. 25015402 10.18632/oncotarget.2151 PMC4170600

[bib48] He TC, Zhou SB, da Costa LT, Yu J, Kinzler KW, Vogelstein B. A simplified system for generating recombinant adenoviruses. Proc Natl Acad Sci USA 1998; 95: 2509–2514. 9482916 10.1073/pnas.95.5.2509 PMC19394

