# Supplementary material for: RGD-modifided oncolytic adenovirus exhibited potent cytotoxic effect on CAR-negative bladder cancer-initiating cells
Source: Cell Death Dis. 2015 May 14;6(5):e1760–. doi: 10.1038/cddis.2015.128 (PMC4669706; doi:10.1038/cddis.2015.128)
Supplement: Supplementary Table S1 [file cddis2015128x1.doc]

**Supplementary Table S1. Sequences of Primers**

| **Primers** | **Sequences** |
| --- | --- |
|  |  |
| WT-forward | 5’-AGAGCCCATGGAACCCGAGA-3’ |
| WT-reverse | 5’-CATCGTACCTCAGCACCTTCCA-3’ |
|  |  |
| TRAIL-forward | 5’-ATGCTTTAAAATGGCTATGATGGAGGT-3’ |
| TRAIL-reverse | 5’-ATCGTTTAAATTAGCCAACTAAAAAGG-3’ |
|  |  |
| RGD-forward-1 | 5’- GCTTGAGGTTAACCTAAGCACT-3’ |
| RGD-reverse-1 | 5’- AGTTGTGTCGCAGAAGCAATCTCCAC-3’ |
|  |  |
| RGD-forward-2 | 5’- GGAAACAGGATGTGATTGTCGTGGAG-3’ |
| RGD-reverse-2 | 5’- AAATGACTTGAAATTTTCTGCAAT-3’ |
|  |  |
| E1A (Δ24) -forward  E1A (Δ24) -reverse | 5’- CCGACTCTGTAATGTTGGCG-3’  5’- CGCCGTTTACAGCTCAAGTCCAA-3’ |
|  |  |
| MRP1-forward | 5’-AAGACCAAGACGTATCAGGT-3’ |
| MRP1-reverse | 5’-CAATGGTCACGTAGACGGCAA-3’ |
|  |  |
| GAPDH-forward | 5’-GTCTCCTCTGACTTCAACAGCG-3’ |
| GAPDH-reverse | 5’-ACCCCCTGTTGCTGTAGCCAA-3’ |
|  |  |
| survivin-forward | 5’-CTAGAAGCTTATGGTGAGCAAGGGCG-3’ |
| survivin-reverse | 5’-ATCGGGATCCTTACTTGTACAGCTCG-3’ |
|  |  |
| Nanog-forward | 5’-AGAAATCCCTTCCCTCGCCA-3’ |
| Nanog-reverse | 5’-TGGTAGAAGAATCAGGGCTG-3’ |
|  |  |
| E3-forward | 5’-TACCGGACTTACATCTACCAC-3’ |
| E3-reverse | 5’-AACATAAGCGCTATGGAGAAC-3’ |
|  |  |
