# Supplementary material for: RGD-modifided oncolytic adenovirus exhibited potent cytotoxic effect on CAR-negative bladder cancer-initiating cells
Source: Cell Death Dis. 2015 May 14;6(5):e1760–. doi: 10.1038/cddis.2015.128 (PMC4669706; doi:10.1038/cddis.2015.128)
Supplement: Supplementary Table S2 [file cddis2015128x2.doc]

**Supplementary Table 2. Efficiency of Infection**

| **Virus name** | **Ct** | **lg(Copy number**  **of gDNA)** | **Copy number of gDNA** | **Efficiency**  **(%)** |
| --- | --- | --- | --- | --- |
| **Onco^Ad^. hTERT-EGFP** | 19.79 | 4.03 | 10765 | 5.38 |
| **Onco^Ad^.RGD-hTERT-EGFP** | 19.23 | 4.67 | 46760 | 23.38 |
| **Onco^Ad^.RGD-hTERT-TRAIL** | 18.48 | 4.69 | 49492 | 24.75 |
