# Supplementary figures and images for: RGD-modifided oncolytic adenovirus exhibited potent cytotoxic effect on CAR-negative bladder cancer-initiating cells
Source: Cell Death Dis. 2015 May 14;6(5):e1760–. doi: 10.1038/cddis.2015.128 (PMC4669706; doi:10.1038/cddis.2015.128)

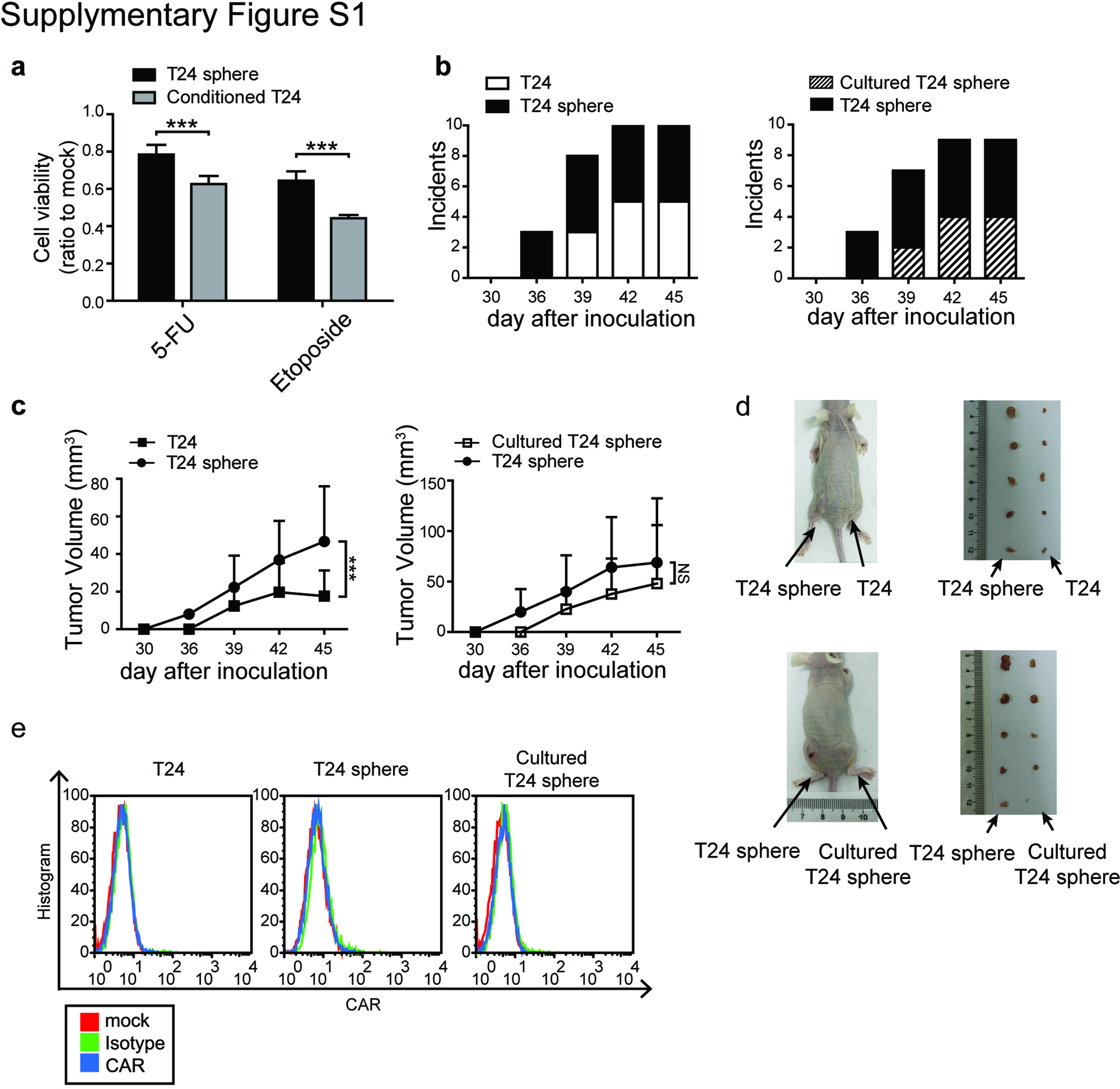

Supplement: Supplementary Figure S1 [file cddis2015128x3.tif]

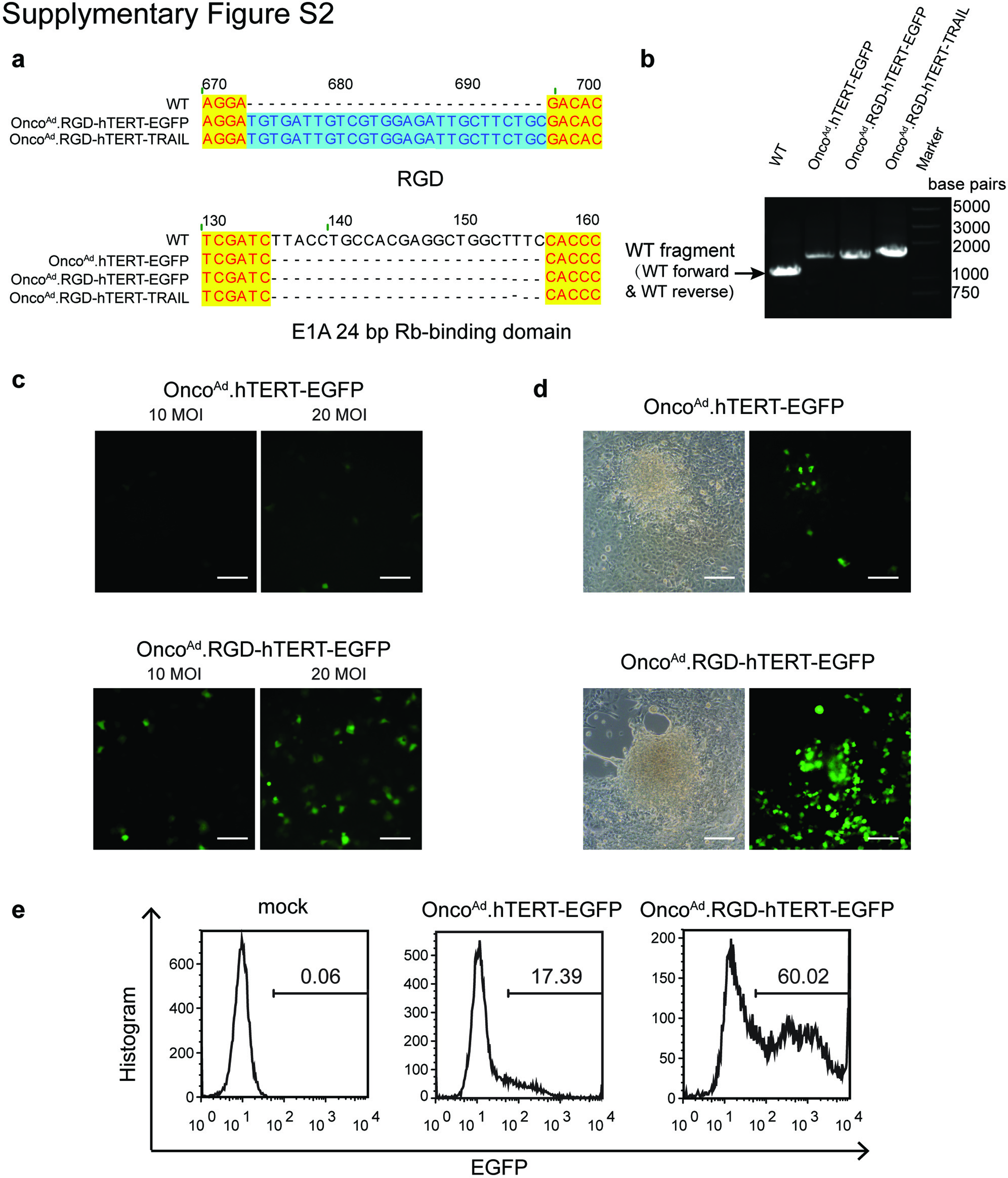

Supplement: Supplementary Figure S2 [file cddis2015128x4.tif]

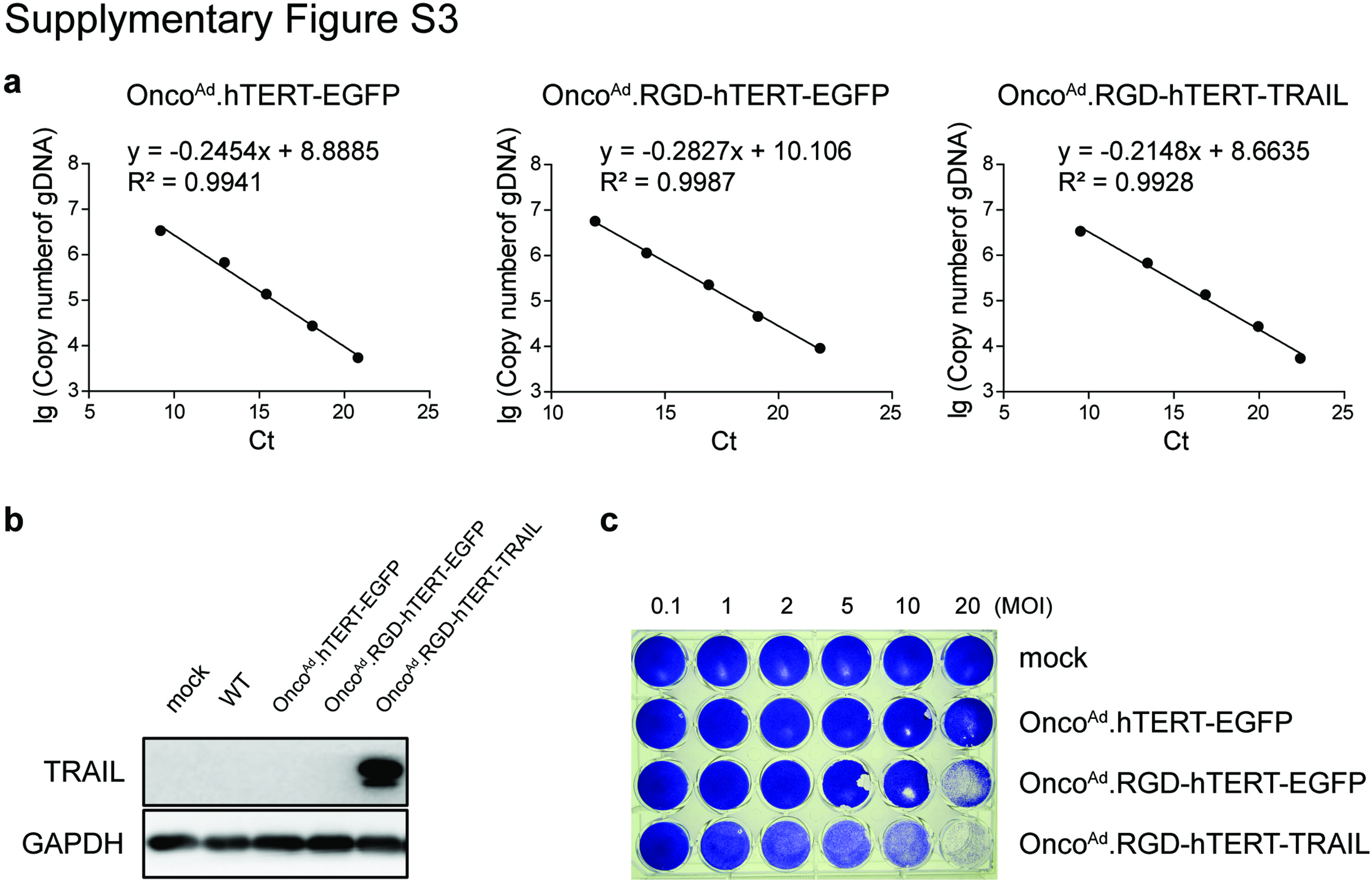

Supplement: Supplementary Figure S3 [file cddis2015128x5.tif]

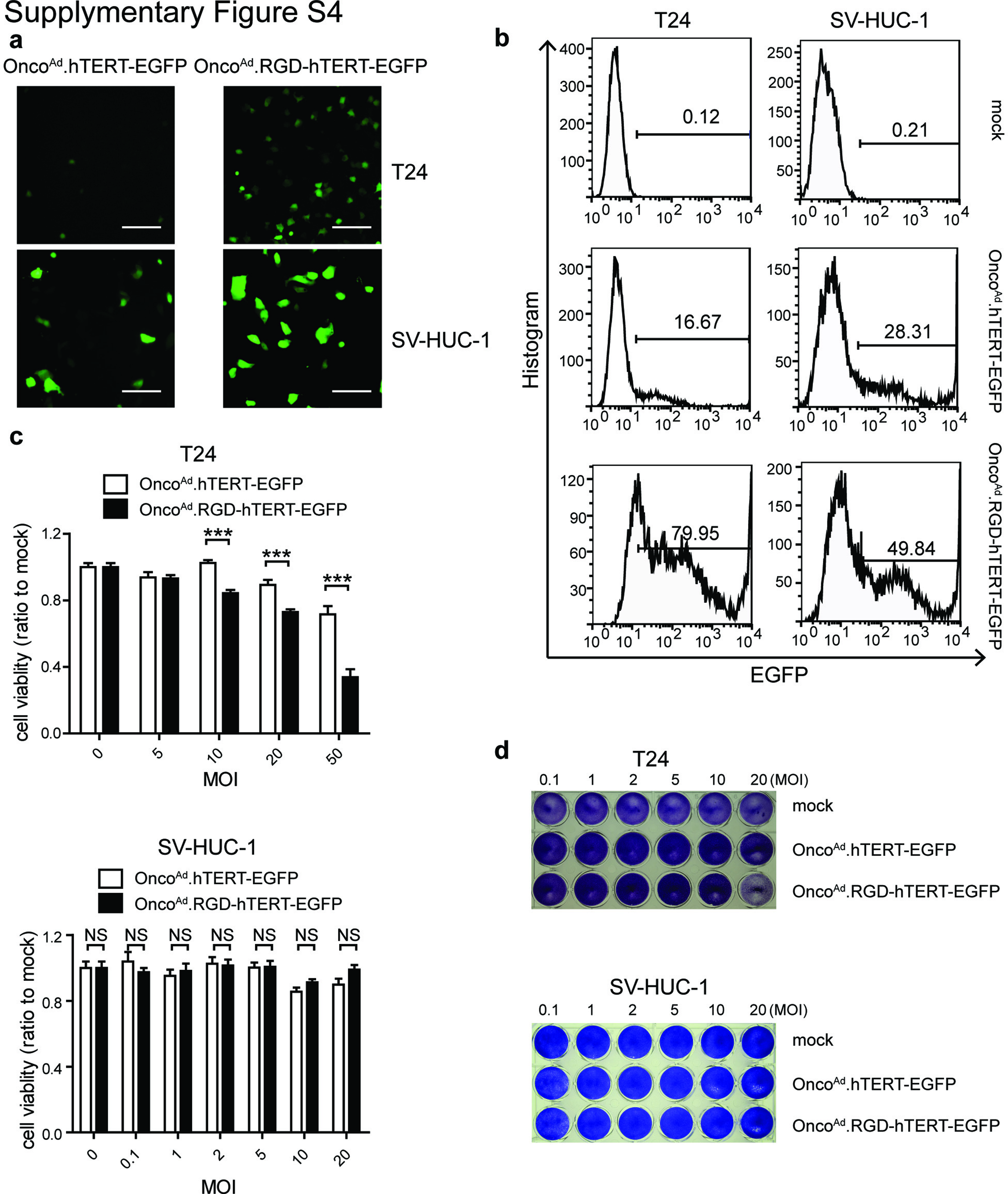

Supplement: Supplementary Figure S4 [file cddis2015128x6.tif]

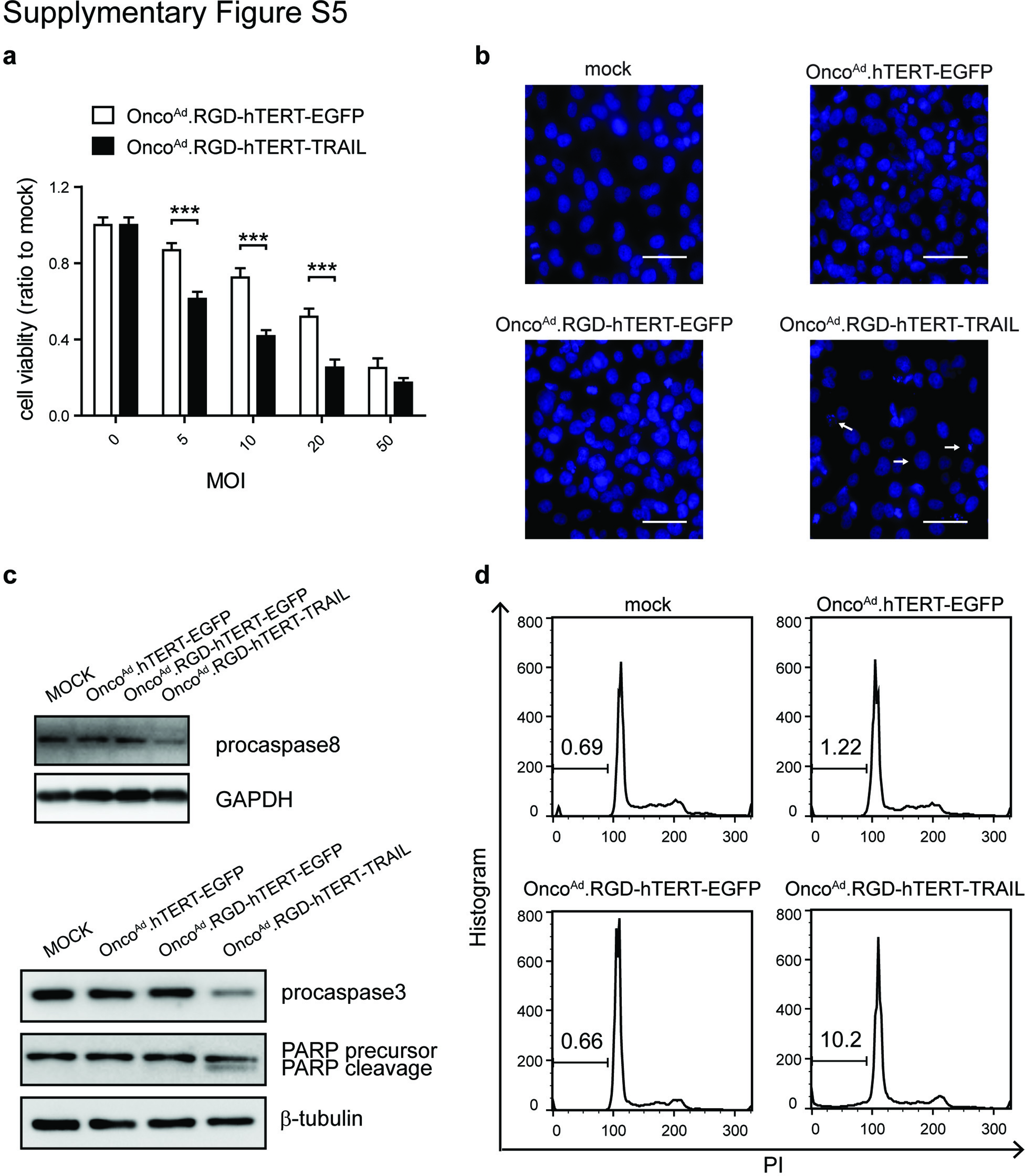

Supplement: Supplementary Figure S5 [file cddis2015128x7.tif]
