# Supplementary material for: RGD-modifided oncolytic adenovirus exhibited potent cytotoxic effect on CAR-negative bladder cancer-initiating cells
Source: Cell Death Dis. 2015 May 14;6(5):e1760–. doi: 10.1038/cddis.2015.128 (PMC4669706; doi:10.1038/cddis.2015.128)
Supplement: Supplementary Figure Legends [file cddis2015128x8.doc]

**Supplementary Figure legends**

Supplementary Figure S1: CAR-negative T24 sphere cells exhibited stronger chemoresistance than Conditioned T24 cells and tumor initiation ability than T24 cells and Cultured T24 spheres cell. 1×10^2^ T24 sphere cells were injected at the left rear of nude mice, and the same number of T24 cells or Cultured T24 sphere cells were injected at the right rear. (a) T24 sphere cells showed stronger stability than conditioned T24 cells after treated with 5-FU (100 μg/mL) and etoposide (10 μg/mL) for 2 days. Cell viability was detected with MTT assay and repeated for three times. The relative cell viability was shown by fold change to the corresponding mock. (b) T24 sphere cells initiated xenografts earlier than T24 cells and cultured T24 sphere cells. Incidence indicated the number of mice with palpable tumor. (c-d) Tumor growth curve and corresponding tumor size of xenografts established by T24 cells, T24 sphere cells and cultured T24 cells. Tumor volume was measured every three days and the data shown represented mean ± SD (n=5). The number of mice in each group were five. ***P<0.001, NS = no significance. (e) CAR expression were barely detected in T24 cells, T24 sphere cells and Cultured T24 sphere cells by FACS analysis. PE conjugated mouse IgG-1 primary antibody was used as isotype control and mock represented cells not incubated with antibodies.

Supplementary Figure S2: Identification of adenoviruses and detection of their infection ability on T24 sphere cells. (a) Sequence blast of oncolytic adenoviruses for RGD insertion and 24 bp deletion verification. (b) E1B 55KD deletion was verified by PCR suggesting no wide type contamination using the WT forward and reverse primers (labeled in Figure 2a); (c-d) More EGFP positive T24 sphere cells were observed after two-day Onco^Ad^.RGD-hTERT-EGFP treatment at indicated MOI than that of non-RGD modified control Onco^Ad^.hTERT-EGFP by fluorescence microscope in both monolayer (c) and spheroid (d) status, scale bar: 200 μm; (e) The proportion of EGFP positive cells were significantly increased in T24 spheroids mixed with Onco^Ad^.RGD-hTERT-EGFP for 2 days than cells with Onco^Ad^.hTERT-EGFP. EGFP positive cells were detected by FACS analysis.

Supplementary Figure S3: Analysis of infection efficiency and cytotoxicity of RGD-modified adenoviruses on T24 sphere cells. (a) T24 sphere cells were infected with Onco^Ad^. hTERT-EGFP, Onco^Ad^.RGD-hTERT-EGFP or Onco^Ad^.RGD-hTERT-TRAIL for 6 h. Standard curves were drawn by lg (copy number of virus genomic DNA) against Ct value of E3 gene in real-time qPCR. (b) TRAIL expression was detected in T24 sphere cells after treated with Onco^Ad^.RGD-hTERT-TRAIL for 2 days at 10 MOI; (c) Onco^Ad^.RGD-hTERT-TRAIL showed enhanced cytotoxicity in T24 sphere cells. T24 sphere cells were treated with indicated MOI (0.1, 1, 2, 5, 10, 20) of Onco^Ad^.hTERT-EGFP, Onco^Ad^.RGD-hTERT-EGFP and Onco^Ad^.RGD-hTERT-TRAIL for 4 days, respectively, and subjected to crystal violet staining for cell viability determination.

Supplementary Figure S4: Analysis of infection and cytotoxicity of RGD-modified adenoviruses on bladder cancer T24 cells and normal uroepithelial SV-HUC-1 cells. (a-b) The proportion of EGFP positive cells were significantly increased in bladder cancer T24 and normal uroepithelial SV-HUC-1 cells with Onco^Ad^.RGD-hTERT-EGFP treatment for 2days than Onco^Ad^.hTERT-EGFP, scale bar: 200 μm (T24) and 100 μm (SV-HUC-1). (c-d) Onco^Ad^.RGD-hTERT-EGFP elicited enhanced cytotoxic effect on T24 sphere cells (treated for 4 days) but not on SV-HUC-1 cells (treated for 6 days). MTT assay and crystal violet staining was utilized for cell viability analysis. All experiments were repeated three times and all data shown represented mean ± SD (n=3). **P<0.01, ***P<0.001, NS = no significance.

Supplementary Figure S5: Onco^Ad^.RGD-hTERT-TRAIL induced T24 cells apoptosis. (a) Onco^Ad^.RGD-hTERT-TRAIL induced advanced cytotoxicity in T24 sphere cells than Onco^Ad^.RGD-hTERT-EGFP, scale bar: 100 μm. (b) Increased nucleic fragmentation (arrow) was observed in T24 sphere cells after two-day treatment of Onco^Ad^.RGD-hTERT-TRAIL (10 MOI) by Hoechst staining. (c) Up-regulation of PARP protein cleavage and down-regulation of procaspase3 and procaspase 8 protein were observed in T24 sphere cells after treatment with Onco^Ad^.RGD-hTERT-TRAIL (10 MOI) for 2 days; (d) Two-day Onco^Ad^.RGD-hTERT-TRAIL (10 MOI) treatment increased Sub-G1 population of T24 sphere cells. Cell viability was detected by MTT assay. All the experiments were repeated three times and all data shown represented mean ± SD (n=3). ***P<0.001.
